# A Simple Method to Simultaneously Detect and Identify Spikes from Raw Extracellular Recordings

**DOI:** 10.3389/fnins.2015.00452

**Published:** 2015-12-02

**Authors:** Panagiotis C. Petrantonakis, Panayiota Poirazi

**Affiliations:** Computational Biology Laboratory, Institute of Molecular Biology and Biotechnology, Foundation for Research and Technology-HellasHeraklion, Greece

**Keywords:** multi-electrode recordings, spike sorting, extracellular signals

## Abstract

The ability to track when and which neurons fire in the vicinity of an electrode, in an efficient and reliable manner can revolutionize the neuroscience field. The current bottleneck lies in spike sorting algorithms; existing methods for detecting and discriminating the activity of multiple neurons rely on inefficient, multi-step processing of extracellular recordings. In this work, we show that a single-step processing of raw (unfiltered) extracellular signals is sufficient for both the detection and identification of active neurons, thus greatly simplifying and optimizing the spike sorting approach. The efficiency and reliability of our method is demonstrated in both real and simulated data.

## Introduction

Multi-electrode arrays comprise one of the most valuable tools for neuroscience research (Buzsáki, 2004). Neural prosthetics (Gilja et al., 2012), Brain Machine Interfaces (BMIs; Schwarz et al., 2014; Moxon and Foffani, 2015), and neurophysiology experiments (de Lavilléon et al., 2015) are some of the areas where multiple electrodes are routinely used to extract neuronal-activity related information. Analysis of that information toward the identification and discrimination of activity events (spikes) from different neurons, termed Spike Sorting (SS; Gibson et al., 2012), is a multi-step, computationally expensive process, thus limiting its application on wireless technologies that would enable experiments under more ecological conditions (Rey et al., 2015).

**Figure 1:**
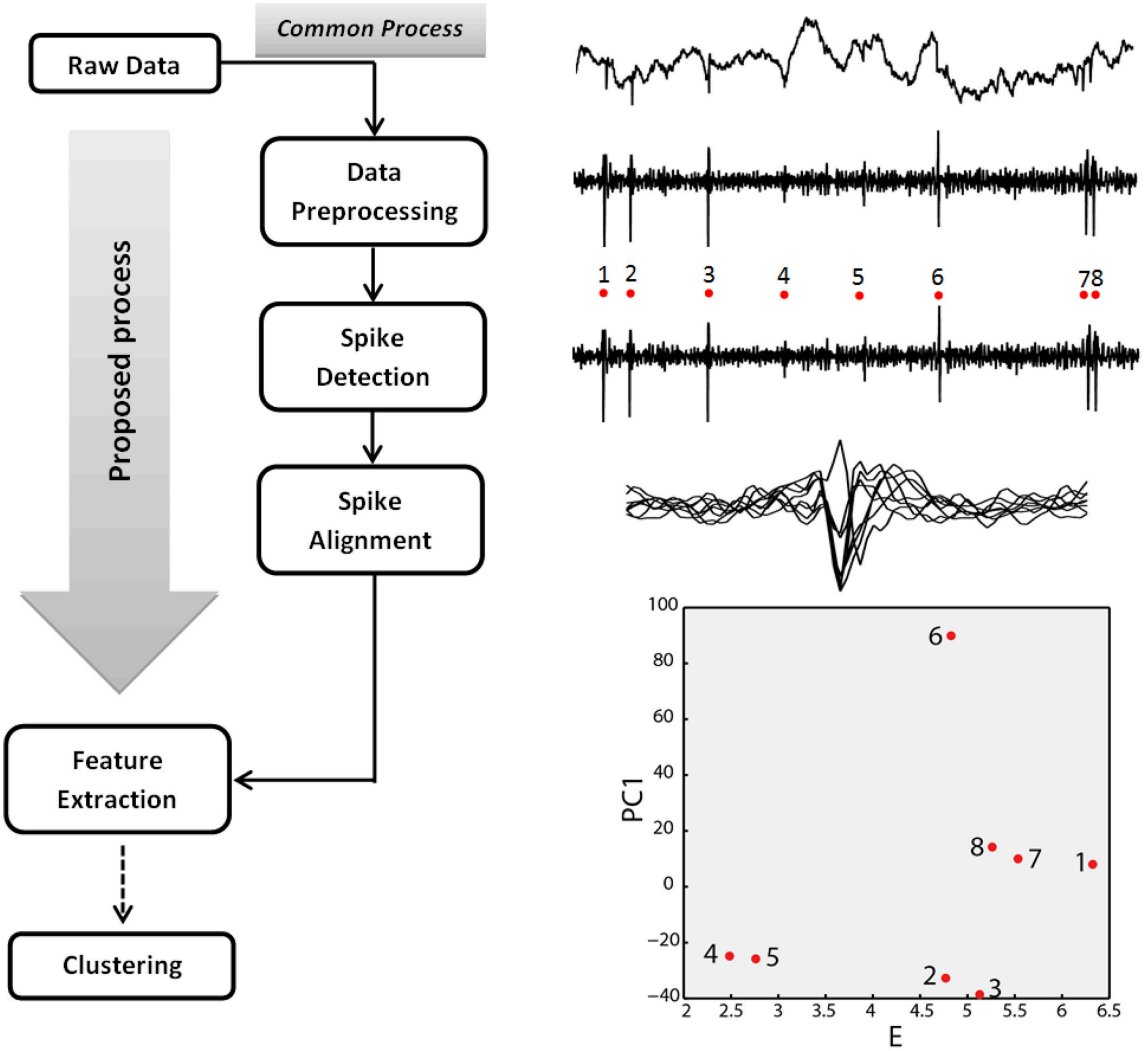
**Spike sorting process**. Left, common and proposed spike sorting processes. Right, Step-by-step illustration of the spike sorting process using part of the 533101 session signal of the real extracellular recordings dataset (Henze et al., 2009; recording channel 3, samples 30501–34000). The depicted example of feature space corresponds to the energy (E) of the aligned spike waveform (abscissa) and the first principal component coefficient (PC1) of the energy normalized spike waveform (Schmitzer-Torbert et al., 2005; ordinate). Spikes 1, 7, and 8 correspond to the same neuron (Henze et al., 2009; intracellularly recorded neuron).

In this work we propose a novel, spike detection and identification algorithm that is applied on raw (unfiltered) extracellular recordings and effectively bypasses several steps of the common SS process. As such, it paves the way for new, wireless on-chip algorithmic implementations, considerably reducing the amount of data that needs to be transmitted through the wireless link. The commonly used state-of-the-art SS process (Einevoll et al., 2012; Figure 1) is divided in five basic steps: (1) the raw extracellular signal derived from a given electrode is band-pass filtered to remove the low frequency component (Local Field Potential) and reveal the spiking activity; (2) spikes are detected; (3) spikes are aligned; (4) specific features are extracted from spikes; (5) data are clustered based on extracted features. Each cluster is assumed to represent a different neuron. In this study, the raw, extracellular signal (derived from a single or multiple electrodes) is processed once and both spike times and discriminatory features are extracted simultaneously (Figure 1).

The proposed algorithm is based on the finding that neuronal spike waveforms are compressible in various basis sets (Thorbergsson et al., 2014). In other words, the spiking waveforms can be decomposed into a set of simpler waveforms, in terms of a coefficient vector, and a support set of few coefficients is sufficient to reliably reconstruct the initial waveform. More importantly, this support set of coefficients is specific for different spike waveforms, namely different neurons (Charbiwala et al., 2011) and obviously different from non-spiking waveforms. Thus, it is suggested that the estimation of, e.g., the euclidean distance, between coefficient sets extracted from consecutive, sliding windows of an extracellular signal would reflect the existence of neuronal spiking. Even better, due to the specificity of the coefficient sets, the estimation of euclidean distance using a sliding window would also discriminate among waveforms originating from different neurons. For instance, the use of a simple basis set of discrete cosines and the decomposition of the signals in a sliding window framework using the Discrete Cosine Transform (DCT; Narasimha and Peterson, 1978) would result in a new signal with the aforementioned properties. Interestingly, since DCT is an orthogonal transformation (thus distance is preserved), the estimation of the new signal can be reduced to a very simple calculation: the euclidean distance between the consecutive windowed signals in their original form would be equivalent to the distance between their decompositions. Our approach is based on this observation.

**Figure 2:**
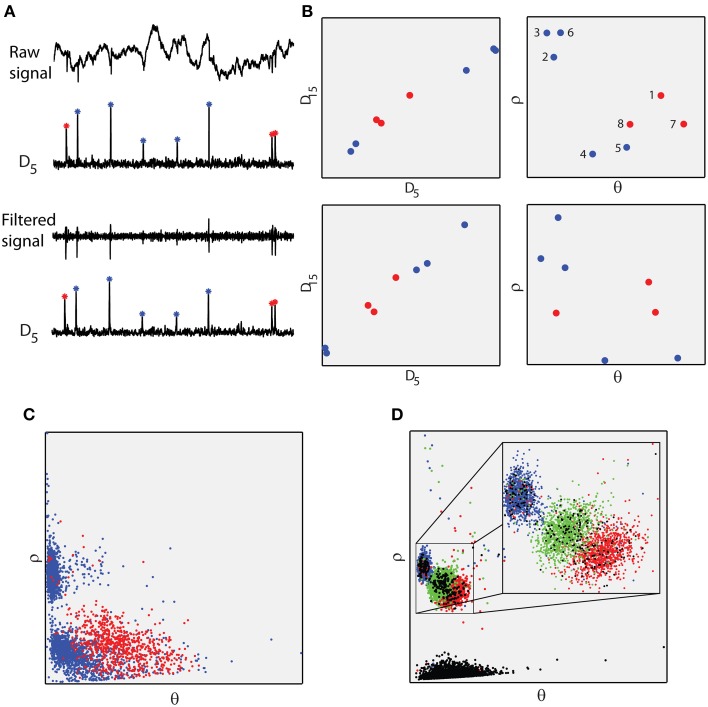
**The proposed features**. **(A)** Raw and filtered signal (same as Figure 1) and corresponding *D*_5_ signals. Maxima of the *D*_5_ signal correspond to spikes in both the raw and filtered recordings. Red stars correspond to the same neuron (Henze et al., 2009; intracellularly recorded neuron). Red stars lay approximately in the same amplitude level, different from the blue ones. **(B)** Left, amplitude of *D*_15_ maxima against amplitude of *D*_5_ maxima, estimated from raw (first row) and filtered (second row) recordings. Right, the corresponding polar coordinates. Polar coordinates of the *D*_5_ and *D*_15_ maxima comprise the proposed feature space. Red dots correspond to red stars in **(A)**. **(C)** Feature space for the entire 533101 raw signal. Red dots correspond to the spiking activity of the intracellularly recorded neuron. Blue dots correspond to “noisy” spiking activity. **(D)** The proposed feature space of dataset 3 in the fourth level of noise from the simulated database (Quiroga et al., 2004). Blue, green, and red dots correspond to three different neurons. Black dots correspond to falsely detected spikes.

The main processing step of the proposed approach is the segmentation of the raw, single/multiple-electrode signal using a sliding, fixed-length window. A new signal, *D*_*w*_, where *w* denotes the length of the window, is produced by estimating the euclidean distances between the different signal segments (see Section Materials and Methods and Supplementary Figure 1). Within the resulting signal *D*_*w*_, the time points of the local maxima indicate the time points of the spiking events while their amplitudes serve as unique markers (identifiers) for each specific spike waveform, namely each distinct active neuron (Figure 2). Strikingly, this is true for both the band-pass filtered and the raw, unfiltered version of the extracellular signal. In essence, different support sets of coefficients for different spike waveforms, lead to different amplitudes of local maxima for *D*_*w*_. Thus with a single processing step, the proposed method transforms the unfiltered, extracellular signal into a new signal (*D*_*w*_) that reveals both the occurrence and identity of spikes in a simple and highly efficient manner.

## Materials and methods

### *D*_*w*_ and feature space extraction

The *D*_*w*_ signal consisted of the Euclidian distance values between the successive *X*_*k*_ signal segments. For instance, $D_{w}\left(1\right)=\sqrt{\overset{w}{\underset{k=1}{\sum }}{\left(X_{k}^{1}-X_{k}^{2}\right)}^{2}}$ where $X_{k}^{1}$ and $X_{k}^{2}$ are the first two successive segments of length *w* using a predefined step. In this work we used *w* = 5 and *w* = 15 as most significant decomposition coefficients varied within this range (Charbiwala et al., 2011). The step value was 1 sample. The *D*_5_ and *D*_15_ signals were used to extract the proposed feature space, which consisted of the polar coordinates of the amplitudes of their local maxima (Figure 2). Polar coordinates instead of Cartesian ones where used in order to avoid the linearity that is exhibited between *D*_5_ and *D*_15_ values (see Figure 2B). Expansion of the methodology to account for tetrode recordings was performed by estimating *D*_*w*_ using four channels simultaneously. Specifically, the *X*_*k*_ segments where concatenated before the euclidean distance calculation, resulting in a single *D*_*w*_ signal that incorporated information from all four recordings (see Supplementary Figure 1B).

### Spike detection

In this work, spike-detection (i.e., the detection of maxima in the *D*_*w*_ signal) and feature-extraction (i.e., amplitudes of maxima in the *D*_*w*_ signal) are essentially a single step, as the maximum values of *D*_*w*_ are detected mainly around the time points of a spike occurrence. The detection of maxima is based on *D*_*w*_ amplitude thresholding, in particular using the *D*_5_ signal. In all examples reported here (real or simulated), the threshold (Quiroga et al., 2004) was set to *T* = 2σ_*n*_ where $\sigma_{n}=median\left\{\frac{\left|D_{w}\right|}{0.6745}\right\}$ is an estimate of the standard deviation of the background noise (Donoho and Johnstone, 1994). A lower threshold could also be used as the thresholding process is applied on the *D*_*w*_ signal and not on the extracellular signal itself and, thus, it would not affect the subsequent clustering process. Moreover, to avoid multiple detections of the same spike, a minimum sample distance between two detected maxima of 15 samples (equal to the maximum window length used) was imposed. Finally, due to the sharper form of maxima peaks in *D*_5_ compared to *D*_15_, maxima detection was first performed in *D*_5_. Afterwards, identification of the maxima amplitude in *D*_15_ (occurring approximately at the same time points as in *D*_5_) was performed using the same time points detected in *D*_5_.

### Datasets

The examples analyzed were taken from two public databases, one consisting of real extracellular recordings and one consisting of simulated signals with integrated spike waveforms. The former is the hc-1 database (Henze et al., 2009) that consists of simultaneous intracellular and extracellular recordings in the CA1 hippocampal region of anesthetized rats. It contains both intracellular and extracellular recordings from the same neuron. Ten different signals, corresponding to 10 different cells, were analyzed from this database. For the single channel analysis the experimental sessions and single recording channels (see Supplementary Figure 2) were selected in order to account for various firing patterns (bursting etc.) and to have a reliable representation of the intracellularly recorded neuron in the extracellular recordings. Some of the recordings were from tetrodes and some from hexatrodes settings with two shanks. In the four-channel analysis that we followed for the estimation of *D*_*w*_, in the case of double hexatrodes, only four channels were used. These were selected based on their distance from the single electrode that was initially chosen for the single electrode analysis, in order to avoid problems that have to do with distances between electrodes (Rossant et al., 2015).

The latter database consists of four different datasets that refer to different levels of difficulty in terms of spike detection and identification (Quiroga et al., 2004), hereafter referred as datasets 1, 2 (easy) and 3, 4 (difficult). Moreover, the four different datasets were constructed using different levels of background noise (Quiroga et al., 2004). The noise level was determined from its standard deviation relative to the amplitude of the integrated spike waveforms. Four levels of noise, associated with four different standard deviation values, i.e., 0.05 (noise level 1 etc.), 0.1, 0.15, 0.2 were used. For dataset 1, four additional levels of noise were applied with deviations equal to 0.25, 0.3, 0.35, and 0.4 (see Supplementary Figure 3), since spike identification was relatively easy (more information about the construction of the simulated data used can be found in Quiroga et al., 2004). It should be noted that simulated data lack, in principle, the low frequency component, i.e., cannot be considered as raw (unfiltered) simulated signal. They are primarily used for further evaluation of the proposed approach for multiple ground truth neuronal activity.

### Classification

**Figure 3:**
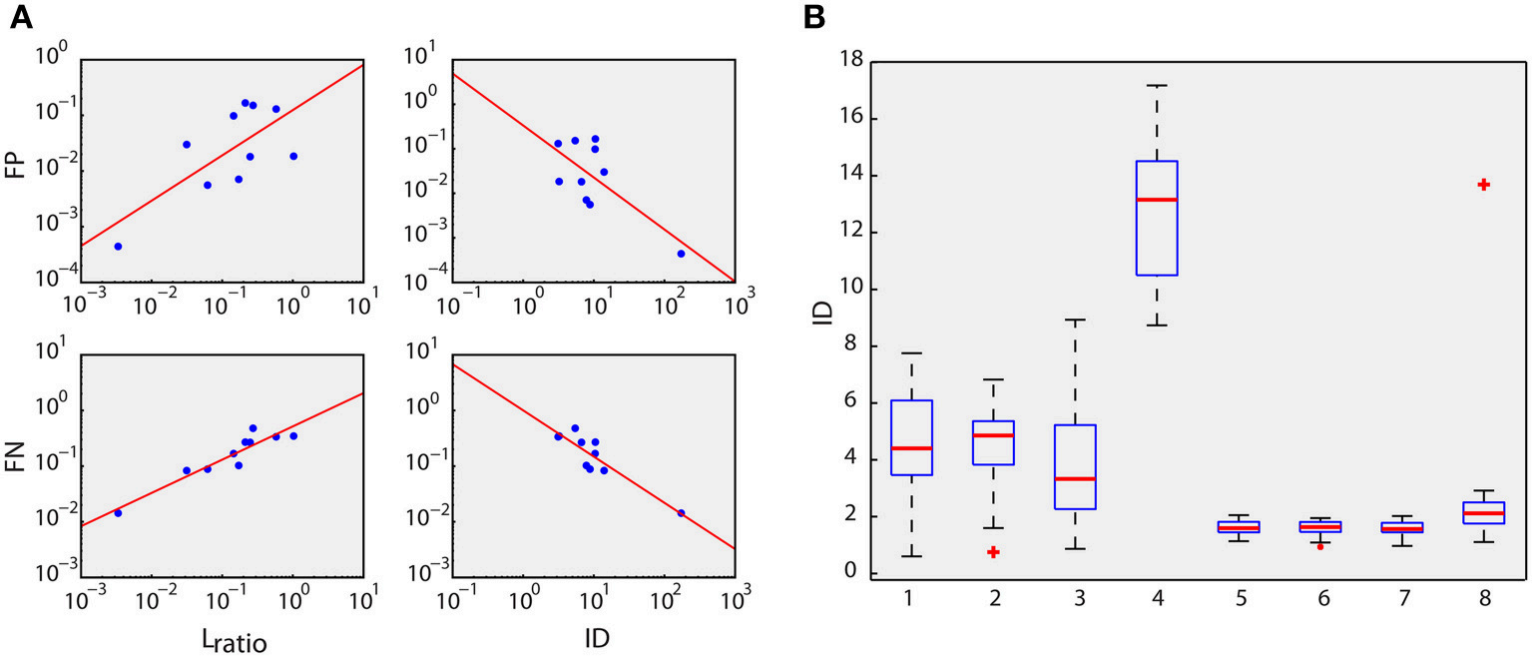
**Feature space evaluations**. **(A)** Type I (False Positives, FP) and type II (False Negatives, FN) error rates against *L*_*ratio*_ and Isolation Distance (ID) measures (Schmitzer-Torbert et al., 2005) in the log scale of 10 different datasets of real data. Linear correlations are significant (Pearson Correlation, *p* < 0.05). **(B)** ID between and within clusters of the simulated dataset (Materials and Methods). Boxplots 1, 2, 3, and 4 depict the IDs of the clusters of neurons 1, 2, 3 and falsely detected spikes (see Figure 2D), respectively, from the other clusters (between-cluster IDs). Boxplots 5, 6, 7, and 8 are the IDs of the clusters of neurons 1, 2, 3, and falsely detected spikes from the corresponding cluster itself (within-cluster IDs). All comparisons (ANOVA) of four pairs of within- and between-cluster IDs, e.g., pair 1 and 5, reveal high statistical significance (*p* ≪ 0.001).

For the classification of spikes corresponding to the intracellularly recorded neuron (hc-1 database) against all other “noisy” spikes (spikes that do not correspond to that particular neuron) the *k*-Nearest Neighbor (*k*-NN) classifier (Cover and Hart, 1967) was used. For each one of the ten different cells (see Supplementary Figure 2) a 100-iteration procedure was implemented. Every time, 50% of the data were randomly selected for training and the rest were used for testing. The mean type I and type II error values were used for the extraction of Figure 3 (see also Supplementary Table 1 for mean classification performance for each one of the different cells). For the *k*-NN algorithm, the number of nearest neighbors was set to *k* = 4.

### *L_ratio_* and isolation distance (ID) estimation

In order to evaluate the capacity of the proposed features to separate the spikes that belong to different neurons we used the *L*_*ratio*_ and the Isolation Distance (ID) measures (Schmitzer-Torbert et al., 2005). Both measures make use of the Mahalanobis Distance (MD; Mahalanobis, 1936). The MD of spike *i* from cluster *C* is estimated using the formula: $MD_{i,C}={\left(x_{i}-\mu_{C}\right)}^{T}\Sigma_{C}^{-1}\left(x_{i}-\mu_{C}\right)$, where *x*_*i*_ is the feature vector for spike *i*, μ_*C*_ is the mean of the feature vector values in cluster *C*, and Σ_*C*_ is the corresponding covariance matrix. The first measure, i.e., *L*_*ratio*_ for cluster *C* is calculated by $L_{ratio}\left(C\right)=\frac{L\left(C\right)}{n_{C}}$ where *n*_*c*_ is the number of spikes in cluster *C*. The quantity *L* for *C* is calculated as $L\left(C\right)=\underset{i\notin C}{\sum }1-CDF_{\chi_{df}^{2}}\left(MD_{i,C}\right)$ where *i* ∉ *C* is the set of spikes which are not members of the cluster *C* and $CDF_{\chi_{df}^{2}}$ is the cumulative distribution function of the χ^2^ distribution with degrees of freedom that equal to the length of the feature vector. A low value of *L* indicates that cluster *C* is well separated from the rest of the data and vice versa.

The ID value of a cluster *C* that contains *n*_*C*_ spikes is defined as the MD value of the $n_{C}^{\text{th}}$ closest noise spike (a spike that does belong to cluster *C*). ID estimates how distant the spikes of cluster *C* are from the other spikes. In Figure 3A the *L*_*ratio*_ and ID values were estimated for the spikes corresponding to the intracellularly recorded cell (cluster *C*). For Figure 3B the ID values were calculated both between clusters and within clusters. In particular, for the boxplots 1, 2, 3, and 4 (see Figure 3B), ID was estimated for spikes that belong to cells 1, 2, 3, and spikes that were falsely detected (black dots), respectively. On the other hand, for boxplots 5, 6, 7, and 8, ID was estimated within the same cluster. For instance, in boxplot 5, the spikes that belong to cell 1 were randomly split into two groups, which consisted of 40 and 60% of the spikes of that cluster, respectively. Subsequently, ID was estimated using the first group (40%) as the reference cluster *C*. This procedure was repeated 10 times and the mean ID was finally used for boxplot 5. The same process was applied for boxplot 6, 7, and 8, i.e., for cells 2, 3, and falsely detected spikes, respectively.

## Results

The proposed analysis was applied both on real (Henze et al., 2009) and simulated (Quiroga et al., 2004) data (see Section Materials and Methods, Figure 2, and Supplementary Figures 2, 3) from single and multiple electrode (tetrode) recordings. Spike detection success rates for single electrode data reached 94.82 ± 9.08 and 92.22 ± 2.64 for real and simulated data, respectively (see Supplementary Tables 1, 2). In both cases, spike detection parameters were identical (see Section Materials and Methods) and the feature vectors consisted of the polar coordinates of the amplitudes of the local maxima in *D*_5_ and *D*_15_ signals (Figure 2 and Section Materials and Methods). Spike detection rates of the proposed method are very similar to those of a complex, multi-step state-of-the-art method (Quiroga et al., 2004) applied on the same simulated data, which demonstrated mean detection rates up to 91.08 ± 6.5 (see Supplementary Table 2). Another important advantage of our method lies in the thresholding applied for spike detection which in our case is done in *D*_*w*_ (see Section Materials and Methods), as opposed to existing methodologies where the thresholding is applied on the filtered extracellular signal. Since detected maxima are subsequently used for clustering, false spike detection due to low thresholds would lead to the formation of additional clusters with very small *D*_*w*_ values that could ultimately be discarded (e.g., see black dots cluster in Figure 2D), thus not influencing the performance of the method and avoiding the process of finding optimum thresholds for spike detection (Rizk and Wolf, 2009).

In addition to detection accuracy, the discrimination power of the extracted features (polar coordinates of the amplitudes of the local maxima in *D*_5_ and *D*_15_) was assessed based on the *L*_*ratio*_ and Isolation Distance (ID) measures (Schmitzer-Torbert et al., 2005). Both measures reveal the degree of isolation of a particular data group (in this case the intracellular recorded neuron, Figure 2C, red dots) from the rest of the data in a feature space (see Section Materials and Methods and Supplementary Figure 4). Their relation with the type I and type II error rates reflects the capacity of the proposed features to isolate spikes from a particular neuron (Schmitzer-Torbert et al., 2005). *L*_*ratio*_ and ID were related linearly on the log scale to type I and type II error rates (see Figure 3A and Section Materials and Methods), confirming the reliability of the proposed features. Type I and II errors were estimated using a *k-NN* classifier (see Section Materials and Methods).

Moreover, we examined whether the proposed feature space assigns spike waveforms of different neurons to discrete and different clusters (see Figure 2D, red, blue, and green dots). Thus, we estimated the ID of each one of the three ground truth clusters of the simulated datasets (Quiroga et al., 2004), firstly, between clusters and then within the same cluster (see Section Materials and Methods and Figure 3B). In all cases tested, ID values within the same cluster were significantly smaller than the ID values estimated between clusters, confirming the initial assumption that waveforms from different neurons have different support sets of coefficients, which is, in turn, reflected in the proposed feature space (*D*_*w*_ maxima).

**Figure 4:**
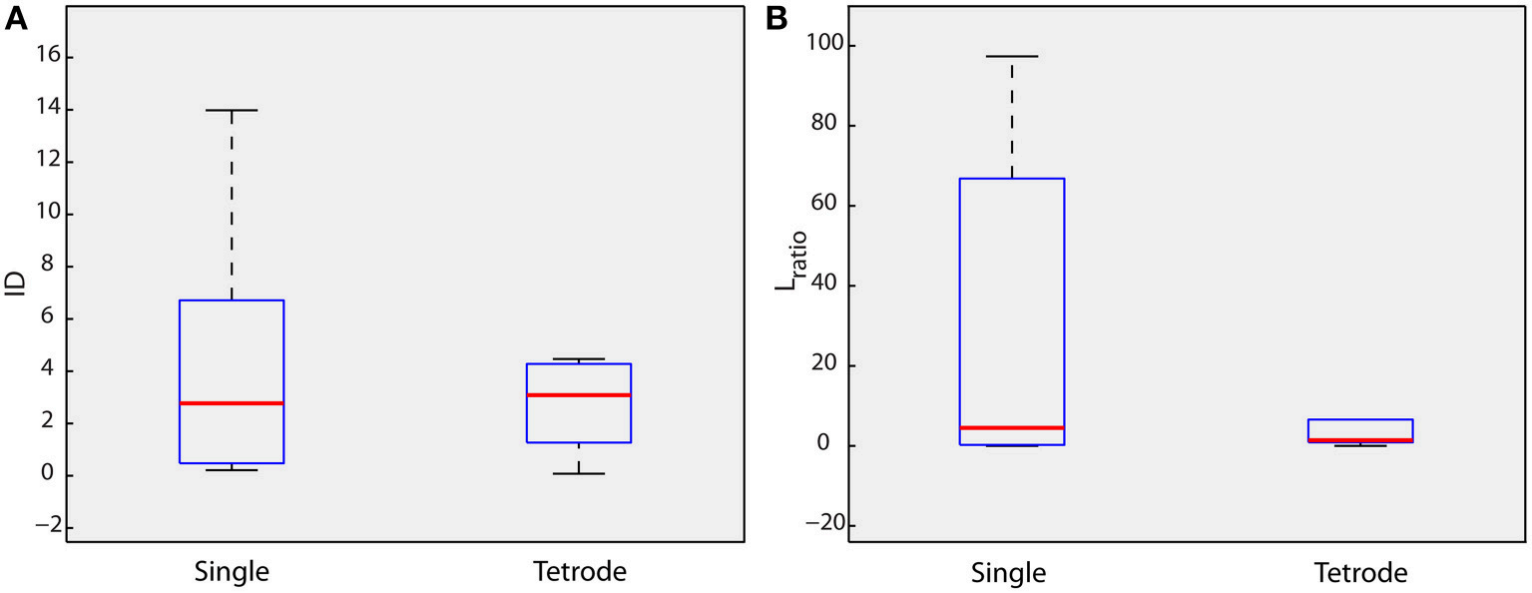
**Tetrode signals**. *L*_*ratio*_ and Isolation Distance (ID) values estimated for the cluster of the intracellularly recorded neuron, for all 10 datasets of real data, when *D*_*w*_ is estimated using only one electrode (single electrode) and four electrodes (tetrode). **(A)** ID **(B)** *L*_*ratio*_.

In the above-mentioned examples, the *D*_*w*_ signal was estimated using recordings from a single electrode. Despite this radical simplification, the method was shown to achieve reliable discrimination of the intracellularly recorded neuron in terms of the ID and *L*_*ratio*_ measures. The proposed methodology however can easily expand to account for multi-electrode experiments. Toward this goal, we reformulated the *D*_*w*_ estimation process to incorporate tetrode signals. Reformulation amounts to a simple concatenation of signal segments corresponding to individual electrodes (see Section Materials and Methods and Supplementary Figure 1B). We then estimated the ID and *L*_*ratio*_ measures of the isolation of the cluster under consideration (intracellular recorded neuron) using the new *D*_*w*_ signal. While median ID and *L*_*ratio*_ values were slightly higher and lower (showing better cluster isolation compared to the single electrode case), respectively, (Figure 4, single: median *ID* = 2.77, median *L*_*ratio*_ = 4.5; tetrode: median *ID* = 3.08, median *L*_*ratio*_ = 1.4) compared to the single electrode analysis, the mean difference was not significant (ANOVA). Importantly, though, the distribution of ID and *L*_*ratio*_ values was narrower (shorter whiskers of boxplots in Figure 4) exhibiting a relevant consistency within the 10 different datasets of real recordings used. Thus, the expansion of the proposed methodology to tetrode signals both slightly enhanced the isolation of the cluster under consideration (intracellularly recorded neuron) and the consistency of the ID and *L*_*ratio*_ values among the datasets.

### Computational complexity

To demonstrate the efficiency of the proposed method, we examined its computational complexity compared to commonly used state-of-the-art-algorithms for spike detection and feature extraction (Gibson et al., 2008). With respect to spike detection (detection of maxima in *D*_*w*_) the computational complexity of our method is equal to that of the most widely used thresholding method for filtered extracellular signals, i.e., 1.058 additions and multiplications per waveform sample (Gibson et al., 2008) which is also the one with the lowest complexity. Note that we by-pass the filtering step as our method is applied on raw signals. With respect to feature extraction, an explicit comparison is not possible as the proposed methodology bypasses several processing steps that would add to the complexity of the ultimate spike sorting algorithm. Nonetheless, the obvious simplistic and straightforward calculation of the *D*_*w*_ makes the proposed approach a very efficient framework in terms of complexity and comprises a significant step closer to wireless on-chip algorithmic implementations.

## Discussion

In sum, we introduced a novel, simple, and efficient approach for simultaneous detection and identification of spikes from raw, unfiltered extracellular recordings. In addition to redesigning the widely used spike sorting process via bypassing several processing steps, the proposed method also overcomes important optimization issues such as the identification of thresholds for spike detection, the scaling to larger sets of recordings and the parallelization of the method. Both scaling and parallelization are straightforward as the *D*_*w*_ signal can be estimated for both single and multiple electrodes using a very similar algorithmic process (see Section Materials and Methods).

In this work we did not propose an unsupervised clustering algorithm for the extracted features. Nevertheless, the cluster isolation analysis revealed the capacity of proposed features to accurately distinguish the activity generated by different neurons. These findings suggest that our methodology and extracted features can be coupled to any clustering algorithm in order to complete the spike sorting procedure. Importantly, for datasets with low noise levels (e.g., simulated datasets, Supplementary Figure 3) cluster discrimination can be achieved using just one of the two polar coordinates, thus further enhancing the efficiency of our method. This is possible because in such low noise cases, even *D*_*w*_ maxima (before polar coordinates estimation) lie in different amplitude levels (identifiers) for different neurons (Supplementary Figure 5), thus, allowing for the implementation for thresholding based clustering algorithms that exhibit significantly low computational complexity and would further support the ultimate goal for on-chip spike sorting implementations.

Future work will be toward that direction, i.e., to investigate the possibility of exploiting the proposed method toward small, wireless, and low-energy consuming recording devices that would either transmit only the time point and the amplitude of the detected maxima in the *D*_*w*_ signal or even perform on-chip, holistic spike sorting realization. Finally, the expansion of the proposed approach to multi-electrode settings (thousands of electrodes) will also be investigated, further diffusing its applicability in contemporary recording settings.

### Conflict of interest statement

The authors declare that the research was conducted in the absence of any commercial or financial relationships that could be construed as a potential conflict of interest.
