# Supplementary material for: A Simple Method to Simultaneously Detect and Identify Spikes from Raw Extracellular Recordings
Source: Front Neurosci. 2015 Dec 2;9:452. doi: 10.3389/fnins.2015.00452 (PMC4667093; doi:10.3389/fnins.2015.00452)
Supplement: Supplementary file 1 [file DataSheet1.DOCX]

Supplementary Material

**A radical approach to simultaneously detect and identify spikes from raw extracellular recordings**

Panagiotis C. Petrantonakis* and Panayiota Poirazi*

*** Correspondence:** ppetrant@imbb.forth.gr, poirazi@imbb.forth.gr

# Supplementary Figures


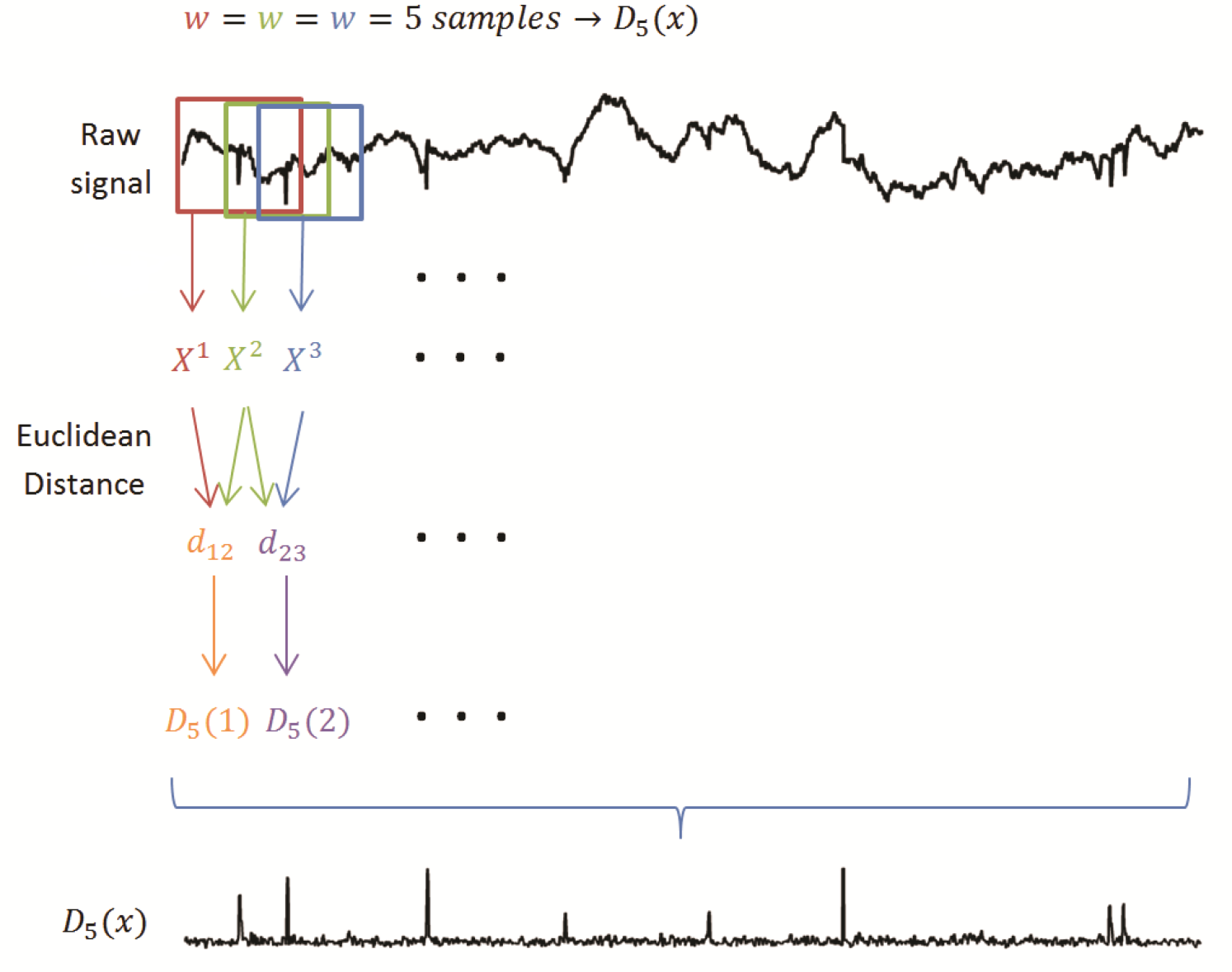


**(A)**

**
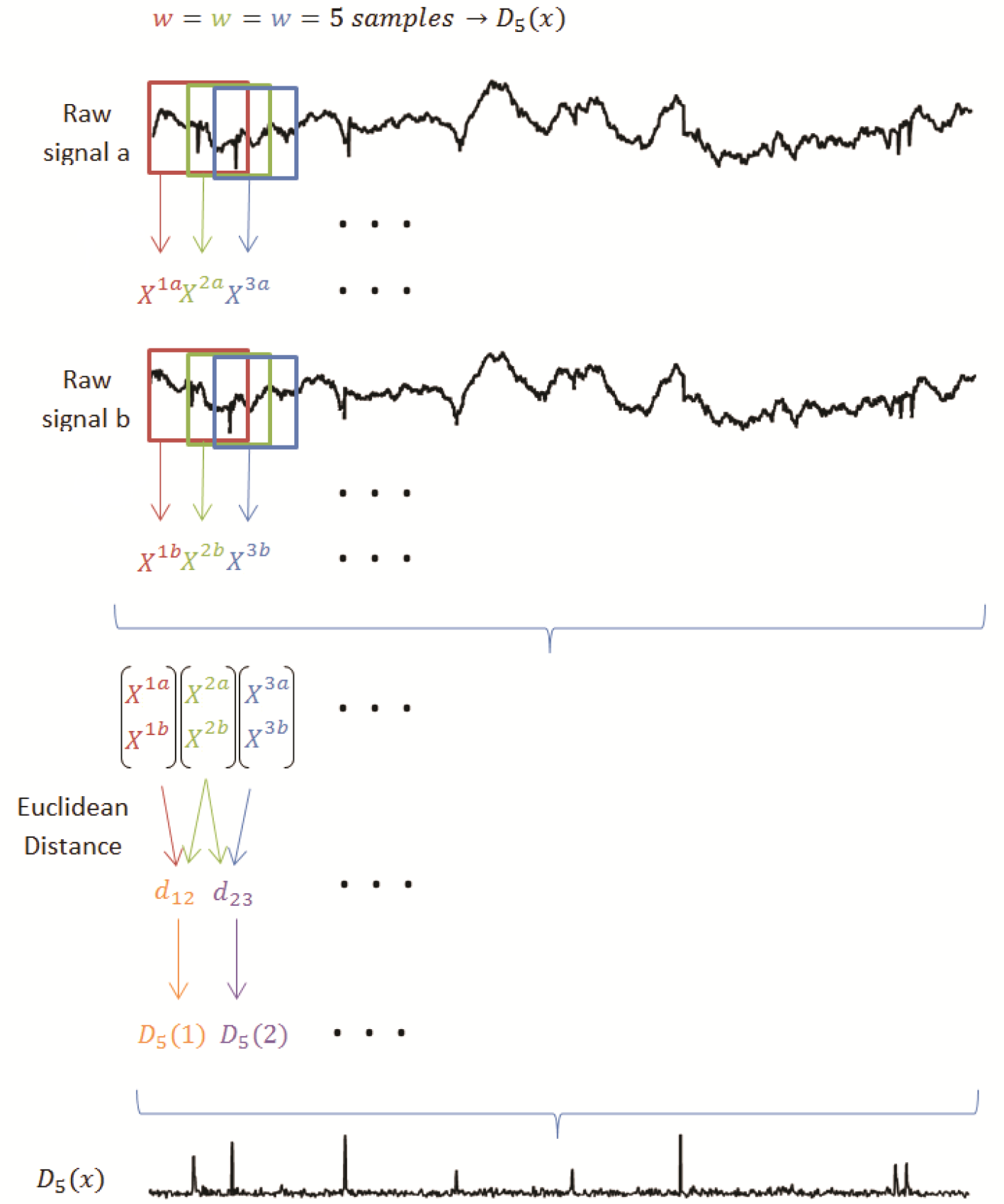
**

**(B)**

**Supplementary Figure 1.** Illustration of the algorithmic procedure for the extraction of $D_{w}$. (A) Consecutive signal parts are extracted from the single-electrode raw signal using a sliding window of length $w$ (Red, green, and blue are three examples of concecutive parts of the raw signal. Their length is constant). After, the respective signal parts $X^{i}, i=1, 2, 3, \ldots$ are extracted, the euclidean distances between them are estimated sequentially. These distances constitute the values of the $D_{w}$ signal. In this example $w=5$. (B) For multiple-electrode case (only two electrodes in this example, a and b), before the euclidean distance calculation, the $X^{i}$ signal parts are concatenated.


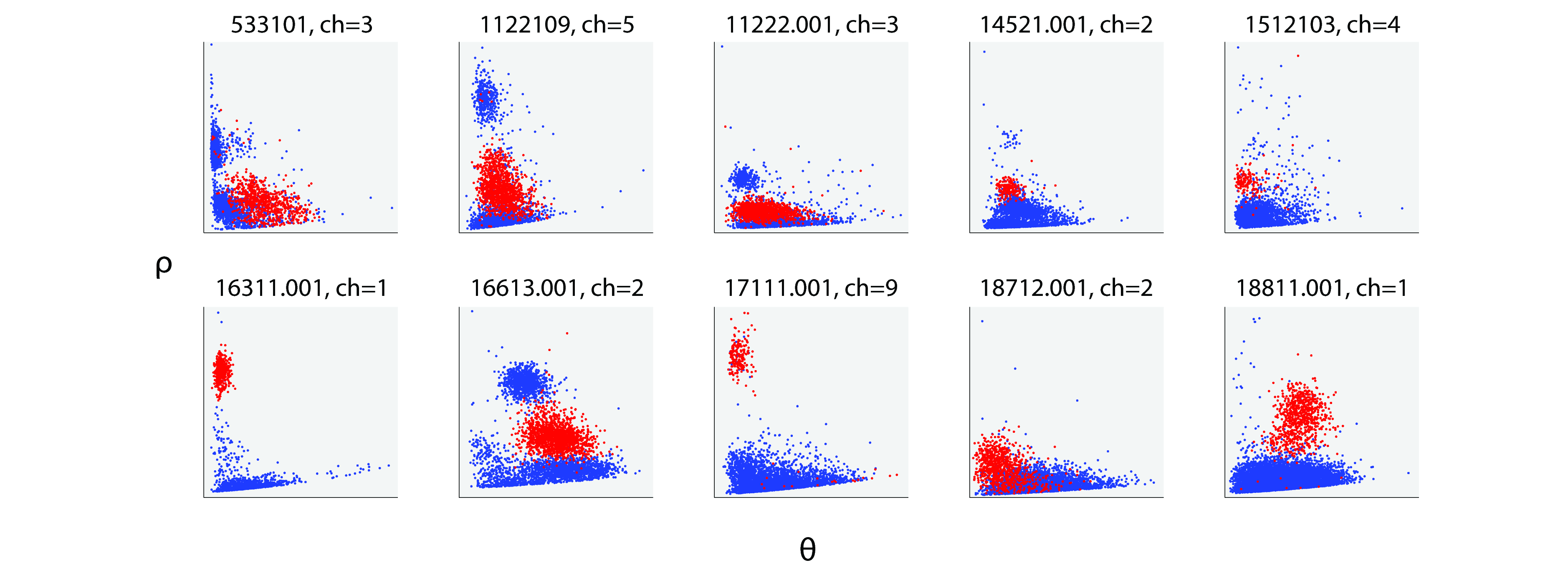


**Supplementary Figure 2.** Feature spaces for the ten real datasets analyzed. Datasets were downloaded from the (hc-1) database. The first subfigure (upper left corner) is the same as Fig. 2c. The rest of the subfigures correspond to the remaining datasets used. The specific experimental session along with the recording channel that was used are depicted on the top of each subfigure.





**Supplementary Figure 3.** Feature spaces for all datasets used from the simulated recordings database. The subfigure that corresponds to the dataset 3 in the 4th level of noise is the same as Fig. 2d (without the spikes –black dots- that are falsely detected). The rest subfigures correspond to the remaining datasets used. Noise levels 5, 6, 7, and 8 were applied only in dataset 1(Quiroga et al., 2004) (see Materials and Methods for noise level description).


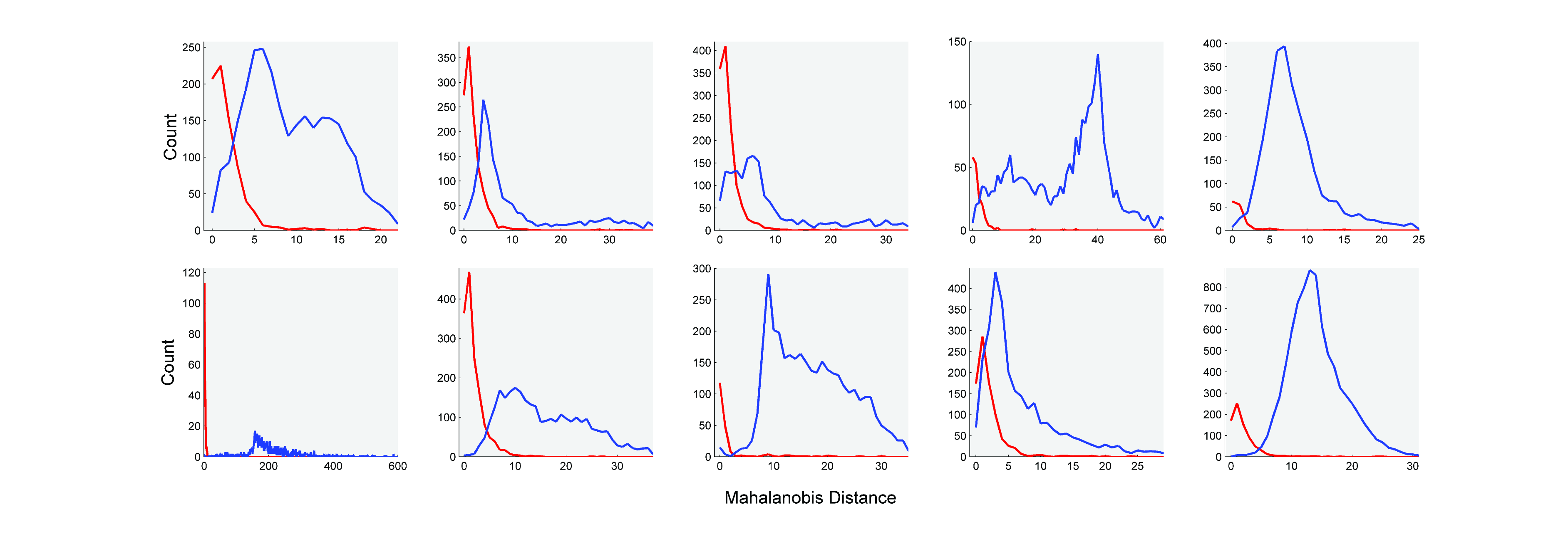


**Supplementary Figure 4.** Mahalanobis Distance distribution for all ten datasets used from the real recordings database. Red and blue lines indicate the distribution of the Mahalanobis Distance (see Materials and Methods) of the red and blue dots of the respective subfigure in Supplementary Figure 2.


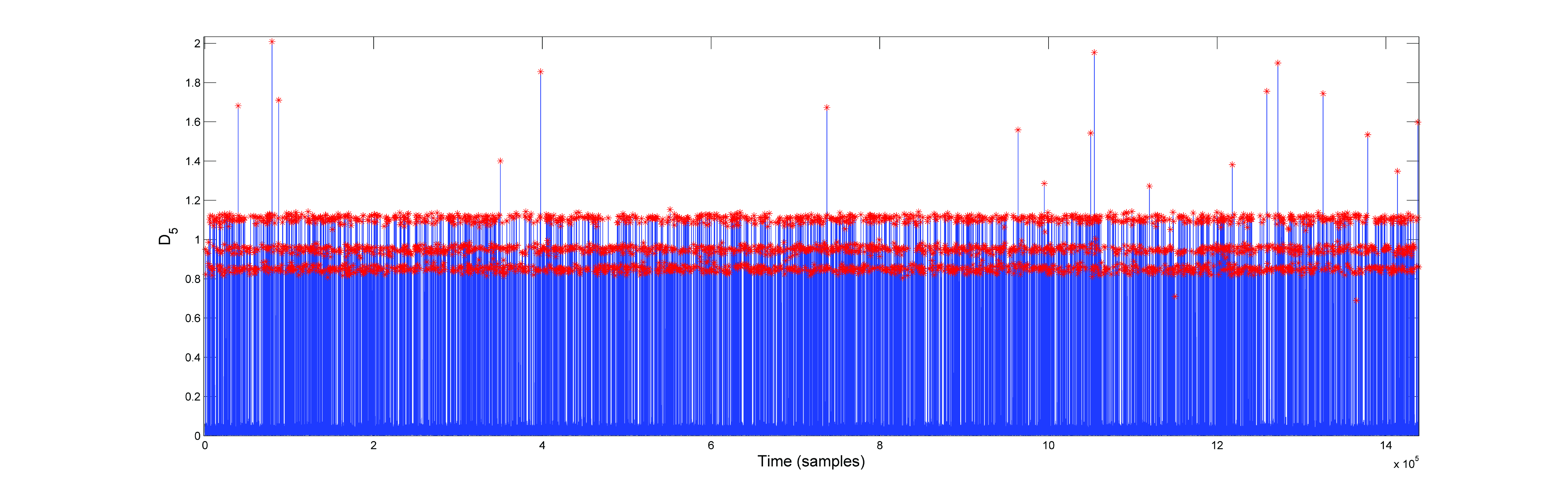


**Supplementary Figure 5.** Signal $D_{5}$ for dataset 3 (noise level 1) of simulated data (see also Supplementary Fig. 3). Red dots indicate the local maxima of the $D_{5}$ signal. Each maxima level corresponds to the activity of one of the three simulated neurons.

# Supplementary Tables

**Supplementary Table 1. Ground truth and detected spikes of each one of the ten datasets from the real recordings database**. Ground truth spikes were estimated using the intracellular recordings. For the detected spikes, the mean classification (spikes of the intracellularly recorded neuron against all other “noisy” spikes) performance is shown for 100 iterations using *k*-NN classifier.

|  | | | | |
| --- | --- | --- | --- | --- |
| Dataset | Ground truth spikes | Detected Spikes | Percentage of detected spikes (%) | Mean Classification performance (%) |
| 533101 | 849 | 770 | 90.69 | 79.85 |
| 1122109 | 1436 | 1196 | 83.29 | 87.16 |
| 11222.001 | 1242 | 1237 | 99.60 | 78.73 |
| 14521.001 | 181 | 181 | 100 | 96.48 |
| 1512103 | 147 | 147 | 100 | 96.52 |
| 16311.001 | 390 | 390 | 100 | 99.57 |
| 16613.001 | 1468 | 1468 | 100 | 95.21 |
| 17111.001 | 200 | 200 | 100 | 98.75 |
| 18712.001 | 1146 | 855 | 74.61 | 83.16 |
| 18811.001 | 786 | 786 | 100 | 98.74 |
| Mean (std): | | | 94.82 (9.08) | 91.42 (8.31) |

**Supplementary Table 2. Ground truth and detected spikes of each one of the datasets from the simulated recordings database**. Detection results from a commonly used state-of-the-art algorithm(Quiroga et al., 2004) are shown for comparison. Detection rates for noise levels 5-8 for Dataset 1were not provided in (Quiroga et al., 2004).

|  | | | | | | | |
| --- | --- | --- | --- | --- | --- | --- | --- |
| Datasets | Noise Level | Ground truth spikes | Detected Spikes (proposed method) | | Percentage of detected spikes (%, proposed method) | Detected Spikes(Quiroga et al., 2004) | Percentage of detected spikes(Quiroga et al., 2004) (%) |
| Dataset 1 | 1 | 3514 | | 3263 | 92.86 | 3304 | 94.02 |
|  | 2 | 3522 | | 3298 | 93.64 | 3343 | 94.92 |
|  | 3 | 3477 | | 3216 | 92.49 | 3117 | 89.65 |
|  | 4 | 3474 | | 3236 | 93.15 | 2485 | 71.53 |
|  | 5 | 3298 | | 3075 | 93.24 | - | - |
|  | 6 | 3475 | | 3129 | 90.04 | - | - |
|  | 7 | 3534 | | 3073 | 86.96 | - | - |
|  | 8 | 3386 | | 2822 | 83.34 | - | - |
| Dataset 2 | 1 | 3410 | | 3176 | 93.14 | 3236 | 94.90 |
|  | 2 | 3520 | | 3263 | 92.70 | 3329 | 94.57 |
|  | 3 | 3411 | | 3200 | 93.81 | 3228 | 94.64 |
|  | 4 | 3526 | | 3303 | 93.68 | 2894 | 82.08 |
| Dataset 3 | 1 | 3383 | | 3158 | 93.35 | 3172 | 93.76 |
|  | 2 | 3448 | | 3238 | 93.91 | 3257 | 94.46 |
|  | 3 | 3472 | | 3243 | 93.40 | 3261 | 93.92 |
|  | 4 | 3414 | | 3190 | 93.44 | 3011 | 88.20 |
| Dataset 4 | 1 | 3364 | | 3098 | 92.09 | 3182 | 94.59 |
|  | 2 | 3462 | | 3255 | 94.02 | 3310 | 95.61 |
|  | 3 | 3440 | | 3180 | 92.44 | 3251 | 94.51 |
|  | 4 | 3493 | | 3240 | 92.76 | 3003 | 85.97 |
| Mean (std): | | | | | 92.22 (2.64) |  | 91.08 (6.50) |

**References**

Quiroga, R. Q., Nadasdy, Z., and Ben-Shaul, Y. (2004). Unsupervised spike detection and sorting with wavelets and superparamagnetic clustering. *Neural Comput.* 16, 1661–1687. doi:10.1162/089976604774201631.
